# Does the structure of inpatient rounds affect medical student education?

**DOI:** 10.5116/ijme.5172.6de1

**Published:** 2013-05-18

**Authors:** Timothy W. Bodnar, Karen E. Fowler, Sanjay Saint

**Affiliations:** 1 Department of Internal Medicine, Division of Metabolism, Endocrinology, and Diabetes, University of Michigan Medical School, Ann Arbor, MI, USA; 2 VA Ann Arbor Healthcare System, Center for Clinical Management Research, Ann Arbor, MI, USA

**Keywords:** Medical students, duty-hour restrictions, work-hour restrictions, teaching rounds, attending rounds

## Abstract

**Objectives:**

To assess whether the organization and structure of inpatient team rounds affects medical student perception of the overall quantity and quality of teaching on an inpatient general medicine service.

**Methods:**

A pilot project to improve inpatient care was launched at the Department of Veterans Affairs Ann Arbor Healthcare System (VAAAHS). General medicine attending physicians involved in the pilot followed a “non-traditional” rounding structure (accentuating senior resident-run “work rounds” while time for “attending rounds” focused on critical issues and teaching). The remainder kept the “traditional” rounding structure (entire team rounds on patients one-by-one). In a cross-sectional design, third- and fourth-year medical students at the University of Michigan were surveyed after their rotation about their experience. Students were asked to rate their educational experience in 21 domains. Responses were evaluated by rounding structure.

**Results:**

A total of 90 students (59%) responded. Across every domain surveyed, students rated the quantity and quality of teaching higher after experiencing “non-traditional” rounds. Statistically significant increases were seen in ratings for “teaching during rounds from senior resident”, “teaching during rounds from attending”, “sit-down teaching from attending”, “overall amount/quality of teaching”, and “overall improvement in internal medicine knowledge”, among others.

**Conclusions:**

The organization and structure of inpatient rounds can significantly impact medical student education. Teaching physicians and medical school clerkship directors should consider this when organizing inpatient team workflow.

## Introduction

Time is a scarce resource in the hospital, and teaching services are no exception. Hospitals and teaching physicians face growing pressure to expedite patient care and focus attention on patient safety and quality, all while managing increasingly complex inpatient care.^1^^,^^2^ Resident physician duty-hour restrictions further limit the time available for teaching.^3^ Clinical teaching of medical students is also affected as medical student duty hours tend to parallel that of residents.^4^ Many worry about untoward effects on education when teaching and rounding time is limited.^3^^,^^5^^,^^6^ Yet this limited time spent with attending physicians is important to medical student education. Medical students perceive "teaching" from residents differently than from attending physicians, notice differences between attendings, and can highlight teaching behaviors that make that difference.^7^^,^^8^ For medical schools and clerkship directors who desire to improve the quantity and quality of medical student inpatient education, finding additional time for teaching may be difficult. Standardizing teaching styles may be even more difficult, given a broad array of attending physician personalities, training, experience, and preferences. However, the organization or “structure” of inpatient rounds may provide an opportunity to standardize and promote behaviors and workflow habits that improve medical student education. The authors sought to evaluate whether a change in rounding structure can affect medical student perception of the overall quantity and quality of teaching they receive on an inpatient general medicine rotation.

## Methods

The University of Michigan Health System Institutional Review Board granted this study “exempt” status (approval # HUM00056642). Research that qualifies for this status includes studying educational methods, surveys/interviews that are anonymous, observation of public behavior, retrospective review of records where the researcher accesses but does not collect identified data, and research with publicly available data that contain personal identifiers. This study qualified for exempt status due to a normal educational setting and voluntary, anonymous surveys.

### Setting

The Medicine Service at the Department of Veterans Affairs Ann Arbor Healthcare System (VAAAHS) in Ann Arbor, Michigan, launched a pilot project in 2009 to improve inpatient care. The Hospital Outcomes Program of Excellence (HOPE) Initiative (www.va-hope.org) “aims to create a stellar inpatient service that could eventually become a national and international model for how inpatient care should be provided in an academic setting.”^9^ Among the most tangible differences for the residents and students rotating with HOPE Initiative attending physicians (one of four VAAAHS Medicine Service teams) is the organization and “structure” of team rounds.The “traditional” model, which is nearly universal among non-HOPE Medicine Service teaching faculty at VAAAHS (as well as among faculty on nearly all inpatient Internal Medicine rotations at the University of Michigan Hospital), emphasizes “pre-rounding” by the interns and students before the attending arrives to conduct rounds with the entire team. Patients are then seen one-by-one, reviewing interval data and discussing all aspects of management. The “non-traditional” model adopted by HOPE Initiative attendings emphasizes “work rounds” run by the senior resident before the attending arrives. This allows the senior resident to review all aspects of management with the interns and students. The team may execute more routine orders earlier (e.g. titration of insulin or supplementation of potassium). “Attending rounds” can then be devoted to more critical aspects of management, allowing time for focused teaching. A comparison schedule for the “traditional” and “non-traditional” structures is shown in Table 1.

**Table 1:** Example of rounding structure and schedule: “traditional” vs. “non-traditional”

Time	“Traditional” Rounds
7:00 _AM_	Students and interns arrive, receive verbal sign-out on their patients from the overnight team
7:15 _AM_	Students and interns “pre-round” on all patients to obtain interval history, perform an exam, and review test results; meanwhile, senior resident is separately reviewing charts and seeing patients
7:50 _AM_	Students and interns quickly discuss major issues with the senior resident
8:15 _AM_	Attending arrives, entire team goes on rounds to see and discuss each patient one-by-one (students and interns present interval data, entire team discusses plan of care, and then reviews with each patient at bedside)
10:30 _AM_	Senior resident goes to Morning Report
Time	“Non-Traditional” Rounds
7:00 _AM_	Students and interns arrive, receive verbal sign-out on their patients from the overnight team
7:15 _AM_	Students and interns join senior resident on “work rounds” on all patients to obtain interval history, perform an exam, review test results, and formulate a plan of care
9:00 _AM_	Attending arrives, reviews selected patients and plans of care with the entire team, and then will see remaining patients either with the senior resident or alone, later reviewing plans of care with the team
10:30 _AM_	Senior resident goes to Morning Report

### Participants

All third-year medical students at the University of Michigan Medical School rotate at the VAAAHS on the Medicine Service (inpatient general medicine wards) for a 4-week period as part of their 12-week Internal Medicine Clerkship. Many fourth-year medical students also choose to rotate for a 4-week sub-internship. Therefore, while all MedicineService teams have 3 or 4 third-year medical students rotating at any given time, each team will have at most 1 fourth-year student and may not have any. All students who rotated on the VAAAHS Medicine Service between October 2010 and June 2011 were eligible for inclusion in this study, and were asked to complete an anonymous, voluntary survey on their educational experience. The only exclusion criterion was declining to complete the survey. Participation was solicited via e-mail at the end of each period, and students were directed to an online survey.

### Survey tool

Our survey was developed expressly for the purpose of this study. Items were chosen to correspond to items on which medical students and teaching physicians (attendings and residents) routinely evaluate each other at the University of Michigan Medical School. The 21 domains rated are delineated in Table 2.

**Table 2:** Medical student responses by rounding structure: “traditional” vs. “non-traditional”

Variable	Traditional N=65	Non-traditional N=25	t	df	p-value
	Mean (SD)	Mean (SD)			
Teaching during rounds from senior resident	5.49 (2.30)	6.72 (2.28)	2.27	88	0.025
Teaching during rounds from your attending	6.88 (2.15)	7.92 (1.75)	2.16	88	0.034
Overall amount/quality of teaching during rounds	6.29 (2.18)	7.68 (1.55)	2.91	88	0.005
Sit-down teaching from your senior resident	5.89 (2.47)	6.76 (2.33)	1.52	88	0.133
Sit-down teaching from your attending	6.85 (2.33)	7.88 (1.69)	2.02	88	0.047
Overall amount/quality of sit-down teaching	6.75 (2.14)	7.72 (1.43)	2.48*	65	0.016
Teaching/reviewing your interviewing skills	3.92 (2.29)	4.57 (2.34)	1.12	81	0.264
Teaching/reviewing your physical exam skills	4.03 (2.47)	4.81 (2.60)	1.23	81	0.222
Teaching/reviewing your note-writing skills	6.48 (2.14)	7.43 (2.27)	1.72	81	0.089
Teaching/reviewing your oral presentation skills	6.92 (2.00)	7.29 (2.37)	0.69	81	0.491
Teaching/reviewing evidence base/primary literature	6.29 (2.38)	7.29 (1.74)	1.76	81	0.082
Overall amount/quality of teaching	6.53 (2.09)	7.86 (1.59)	2.65	81	0.010
Overall improvement in your Internal Medicine knowledge	7.47 (1.60)	8.71 (1.15)	3.30	81	0.002
Overall improvement in your interviewing skills	5.95 (1.95)	6.86 (1.46)	1.95	81	0.054
Overall improvement in your physical exam skills	5.84 (2.09)	6.52 (1.69)	1.36	81	0.178
Overall improvement in your note-writing skills	7.47 (1.81)	8.24 (1.41)	1.78	81	0.080
Overall improvement in your oral presentation skills	7.46 (1.80)	8.43 (1.12)	2.87*	56	0.006
Overall improvement in your evidence-based medicine skills	6.37 (2.07)	7.19 (1.33)	2.09*	55	0.041
Overall quality of the educational experience as a whole	7.58 (1.86)	8.62 (1.12)	3.06*	58	0.003
Your perception of the team’s focus on your education	6.85 (2.22)	8.10 (1.76)	2.32	81	0.023
Amount of “down-time” you had (when residents were busy)	5.27 (2.41)	5.62 (2.50)	0.56	81	0.576

*Satterthwaite test for unequal variances

Note: All responses were rated on a scale of 1 = Worst/Least to 10 = Best/Most.

### Study procedures

Students were not asked to identify their team, attending physician, or other team members. Rather, they were asked only to categorize the team/rounding structure they experienced:

- Traditional rounding structure: Students and interns pre-round on their patients, then meet with the senior resident and attending to discuss, see, and examine each patient one-by-one.
- Non-traditional rounding structure: Students and interns may or may not pre-round on their patients, but will join the senior resident (without the attending) for work rounds to discuss, see, and examine each patient, and then afterwards will meet with the attending (who may or may not see/discuss each patient in detail with the entire team present).

Subsequently, students were asked to rate 21 aspects of their educational experience (see full list in Table 2) on a scale of 1-10, where 1 represented the worst/least, and 10 represented the best/most. This included questions about overall quantity and quality of teaching from the senior resident and the attending during rounds and outside of rounds, questions about teaching and reviewing various skills, and ratings of their own improvement in pertinent knowledge and skill domains. The survey concluded with the option to provide written comments on their educational experience at the VAAAHS. No data was collected on gender or other demographic data from survey respondents. However, note that the University of Michigan Medical School classes are typically comprised of approximately 50-52% men and 48-50% women.

### Statistical analysis

Data was collected online via software utilized at the University of Michigan, and analyzed via SAS statistical software. Descriptive statistics (means and frequencies) were calculated for all variables. Student’s T-tests were conducted to compare responses of those with a “traditional” vs. “non-traditional” rounding structure. Because not all the respondents answered all the questions, the sample size may vary for different results. A P-value of less than 0.05 was considered statistically significant.

## Results

### Survey response rate

A total of 90 out of 152 students completed the survey for a 59% response rate. Of the 152 total students, 39 rotated on a “non-traditional” team, so response rates by rounding structure were 64% (25/39) for “non-traditional” and 58% (63/113) for “traditional”. Seventy-nine (88%) were third-year medical students and 11 (12%) were fourth-year medical students. Sixty-five (72%) rotated on a team that followed the “traditional” rounding structure, while 25 (28%) rotated on a team using the “non-traditional” rounding structure.

### Student perception of quality and quantity of various teaching domains

The mean score on the 10-point Likert scale was higher (better) in the “non-traditional” group for every question (Table 2). The difference was statistically significant (p<0.05) for 11 of the 21 domains surveyed. Students perceived better teaching during rounds, as the average score for quantity and quality of “teaching during rounds” was higher for “non-traditional” (Mean=7.68, SD=1.55) than “traditional” rounds (Mean=6.29, SD=2.18), as was “teaching during rounds from your senior resident” (Mean=6.72, SD=2.28 vs. Mean=5.49, SD=2.30), t _(88)_=2.27, p=0.034, and “teaching during rounds from your attending” (Mean=7.92, SD=1.75 vs. Mean=6.88, SD=2.15), t_(88)_=2.16, p=0.034.Similar statistically significant gains on teams conducting “non-traditional” rounds were seen with overall quantity and quality of sit-down teaching, sit-down teaching from attendings, overall quantity and quality of teaching, improvement in Internal Medicine knowledge, improvement in oral presentation skills, improvement in evidence-based medicine skills, overall educational experience, and perception of team focus on medical student education (Table 2).

## Discussion

University of Michigan Medical School students consistently rated both the quality and quality of teaching higher when rotating on a general medical ward team utilizing a “non-traditional” structure for inpatient rounds. Furthermore, similar gains were seen in the medical students’ perceptions of their own improvement in a variety of knowledge and skill domains. Although not every question showed statistical significance, the trend is clear. This demonstrates that a change in rounding structure can affect the medical student educational experience, and in our particular case, the changes affected that experience in a positive manner.

What may account for this across-the-board improvement? The authors hypothesize that the new rounding structure creates an environment in which all learners achieve an increase in autonomy without the loss of attending supervision. Fostering autonomy in a structured, supportive, and safe way is thought to improve learners’ intrinsic motivation and academic performance.^10^ In the “non-traditional” model, rather than having the students and interns function solely as reporters (regurgitating the information discovered on “pre-rounds” to allow the senior resident and ultimately the attending to make decisions), learners are able to play a more active role during “work rounds.” This may allow students to hone their presentation and medical decision-making skills in a less stressful environment before “attending rounds.” The senior resident still oversees all patient care, as in the “traditional” model, and the attending physician will still see all patients (albeit not always with the entire team present) and discuss all management with the senior resident, if not the entire team.

The “non-traditional” model also may increase the value of the limited time spent on rounding and teaching. Students still gain the worthwhile experiences of “pre-rounding” on their patients, practicing their oral presentation skills, and honing their medical-decision-making skills. “Attending rounds” can then be spent on critical management issues and key learning points on certain patients (rather than, for example, discussing with the attending how much supplemental potassium the patient requires).

Additionally, arriving later may allow time for the attending to better prepare focused teaching points, instead of trying to teach spontaneously during rounds. Furthermore, comments obtained from respondents support the notion that students feel more valued when allowing for this separation between time with the senior resident and time with the attending. As an example, one medical student on a team using the “non-traditional” rounding structure stated:

“I really liked having to pre-round with my senior resident before presenting to the attending. It was probably more helpful than rounding with the attending because during formal rounds, the attending has to cater to everyone, so sometimes students don’t get to present or sometimes are left by the wayside.”

Response rates were similar between the two groups. Our overall survey response rate was only 59%, but this is in line with established acceptable response rates to academic surveys.^11^ The authors believe that the response rate reflected the voluntary, anonymous nature of the survey. No reward or reimbursement was offered.Our study is limited by the lack of randomization of attending physicians. Assignment of residents to ward teams is generally random, as their team assignment is performed by the chief medical residents and is largely based on how day-off requests and previously-scheduled continuity clinics fit into the admitting cycle. Medical students are similarly assigned by administrative staff. Specific team, specific attending, and the HOPE Initiative do not play any role in team assignment for learners. However, there were 9 attending physicians at the VAAAHS who were part of the larger HOPE Initiative (focusing on improving patient safety and quality of care) during the study period. This raises the possibility that the improvement seen in medical students’ ratings was due to the attending rather than rounding structure. However, improvement was seen in every domain (albeit not all were statistically significant). Furthermore, improvement was seen in specific domains that are much more likely affected by the way a team organizes its time than by the direct teaching ability of the attending physician (e.g., “sit-down teaching from your senior resident”). Finally, both the HOPE Initiative faculty and the non-HOPE faculty include attending physicians with a wide range of teaching experience (from first-year faculty to senior staff). Both groups include highly regarded teachers and winners of numerous teaching awards. Therefore, the authors feel it is unlikely that the improvements seen can be explained entirely by a higher quality of attending physicians within the HOPE Initiative. It is possible that participating in the HOPE Initiative provided students with a more positive environment and attitude. However, the authors feel this would not account for improvements in domains that reflect time organization rather than teaching ability or attitudes.Another limitation is that this is a single-center study, evaluating medical student experiences on a general medicine ward service. Results may not be generalizable to all institutions or to subspecialty services. However, the authors propose that the most important point is that the way in which time and responsibility are structured on inpatient rounds provides an opportunity to improve teaching quality and quantity.Future research directions may include expanding on prior qualitative research studies^7^ to better identify the specific aspects of rounding structure that affect medical student education, evaluating the impact on resident physicians, and correlating rounding structure with harder educational outcomes such as performance on standardized exams.

## Conclusions

This study demonstrated that a change in the organization and structure of rounds can significantly impact medical student education. Teaching physicians and medical school clerkship directors should consider how the time devoted to rounds is structured when organizing inpatient team workflow.

## 

### Conflict of Interest

The authors declare that they have no conflict of interest
